# From squiggle to basepair: computational approaches for improving nanopore sequencing read accuracy

**DOI:** 10.1186/s13059-018-1462-9

**Published:** 2018-07-13

**Authors:** Franka J. Rang, Wigard P. Kloosterman, Jeroen de Ridder

**Affiliations:** Department of Genetics, Center for Molecular Medicine, University Medical Center Utrecht, Utrecht University, 3584 CG Utrecht, The Netherlands

## Abstract

Nanopore sequencing is a rapidly maturing technology delivering long reads in real time on a portable instrument at low cost. Not surprisingly, the community has rapidly taken up this new way of sequencing and has used it successfully for a variety of research applications. A major limitation of nanopore sequencing is its high error rate, which despite recent improvements to the nanopore chemistry and computational tools still ranges between 5% and 15%. Here, we review computational approaches determining the nanopore sequencing error rate. Furthermore, we outline strategies for translation of raw sequencing data into base calls for detection of base modifications and for obtaining consensus sequences.

**Electronic supplementary material:**

The online version of this article (10.1186/s13059-018-1462-9) contains supplementary material, which is available to authorized users.

## Introduction

The nanopore sequencing concept was first proposed in the 1980s and has been developed and refined over the past three decades (reviewed in [1]). Rather than the commonly used sequencing-by-synthesis approach, nanopores directly sense DNA or RNA bases by means of pores that are embedded in a membrane separating two compartments. An electric potential is applied over the membrane, resulting in an ion current and flow of DNA through the pore. Nucleotides in the pore change the ion flow, causing distinct current signals that can be used to infer the DNA sequence.

In 2014, Oxford Nanopore Technologies (ONT) released the MinION as the first commercially available nanopore sequencing device. MinION nanopore sequencing offers several advantages over short-read sequencing technologies such as the Illumina MiSeq (Additional file 1: Table S1). First, the MinION produces reads in real time from single molecules. In combination with rapid library preparation, this dramatically shortens the time between sample collection and data analysis. Moreover, the MinION can also be used for direct RNA sequencing without prior reverse transcription or amplification [2]. Second, DNA molecules of any length can be sequenced and reports have been made of reads longer than 800 kb [3] and even exceeding 2 Mb [4]. Long reads are extremely valuable because they provide information on how distal sequences are spatially related. Consequently, they ease genome assembly and structural variant detection [3, 5]. Finally, the MinION is a lot smaller and cheaper than the current short-read platforms, enabling sequencing outside the traditional laboratory context [6, 7]. The key features and applications of MinION sequencing have previously been reviewed by Jain et al. [8]. Following the introduction of the MinION, ONT has commercially released the GridION, which is essentially one instrument with slots for five MinION flow cells and an integrated compute module for base calling. In addition, the PromethION, a high-throughput nanopore platform, is currently being tested by early-access users.

**Fig. 1:**
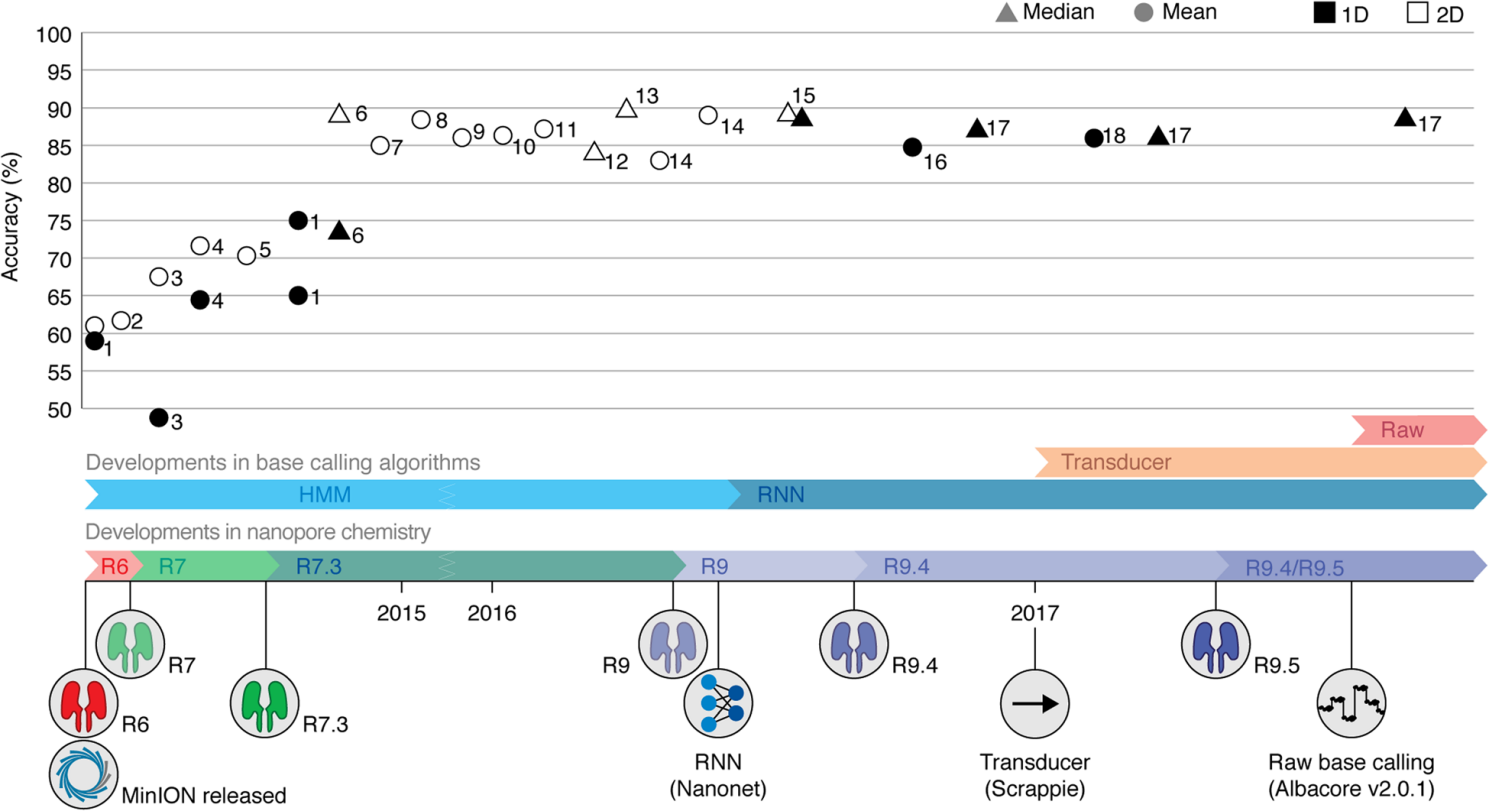
Timeline of reported MinION read accuracies and Oxford Nanopore Technologies (ONT) technological developments. Nanopore chemistry updates and advances in base-caller software are represented as colored bars. The plotted accuracies are ordered on the basis of the chemistry and base-calling software used, not according to publication date. Based on data from 1 [9]; 2 [10]; 3 [50]; 4 [51]; 5 [33]; 6 [28]; 7 [52]; 8 [53]; 9 [54]; 10 [29]; 11 [31]; 12 [48]; 13 [46]; 14 [55]; 15 [11]; 16 [5]; 17 [13]; 18 [3]. HMM Hidden Markov Model, RNN Recurrent Neural Network

A major limitation of MinION sequencing is its lower read accuracy when compared with short-read technologies. When the MinION was first introduced, reads showed an accuracy of less than 60% [9, 10]. This accuracy has improved over recent years to reach approximately 85% [3, 5, 11, 12] (Fig. 1), similar to that of the long-read sequencing technology of PacBio (Additional file 1: Table S1), but still falls short of the more than 99% accuracy offered by short-read platforms. The advantages of long reads outweigh the low-read accuracy for some applications, such as structural variant detection [5]. Furthermore, consensus sequences can be obtained from homogenous DNA samples by (genome) assembly, resulting in accuracies of more than 99% [13–16]. However, the MinION’s low-read accuracy complicates the analysis of complex samples for detection of single nucleotide variations (SNVs) or indels. Successful SNV genotyping based on nanopore reads has been demonstrated [17], but MinION-based SNV calling requires relatively high-coverage sequencing of the variation, for example through targeted sequencing [3, 6, 7, 18].

Since the first release of the MinION, the error rate has considerably improved due to changes in sequencing chemistry. The first MinION flow cells made use of a nanopore called R6, which provided mediocre accuracy. ONT has revealed that the current pore versions (R9.4 and R9.5) are derived from the *Escherichia coli* Curlin sigma S-dependent growth (CsgG) pore [19, 20], and achieve greatly reduced error rates (Fig. 1).

ONT has further improved accuracy by offering the possibility of sequencing both template and complementary strands to obtain a more accurate consensus read. When the double-stranded DNA (dsDNA) is recruited to the nanopore, a motor protein unzips the double strand and passes a single strand through the pore, giving rise to a so-called 1D read (Additional file 1: Figure S1A). Early versions of MinION sequencing offered 2D sequencing involving the reading of both strands, which was enabled by ligation of a hairpin to the DNA (Additional file 1: Figure S1B). The accuracy of 2D consensus reads has generally been more than 5% higher than the accuracy of the template (1D) read alone (Fig. 1). Recently, 2D sequencing was replaced by a new approach termed 1D^2^, which enables the sequencing of template and complementary strands without physical ligation (Additional File 1: Figure S1C). According to ONT, 1D^2^ sequencing can be successful for up to 60% of DNA molecules, and the resulting consensus sequences reach a modal (i.e., most commonly observed) accuracy of ∼ 97% compared with the ∼ 90% accuracy of the 1D reads alone [21, 22]. Research by independent investigators will have to show whether the 1D^2^ chemistry lives up to this promise.

**Fig. 2:**
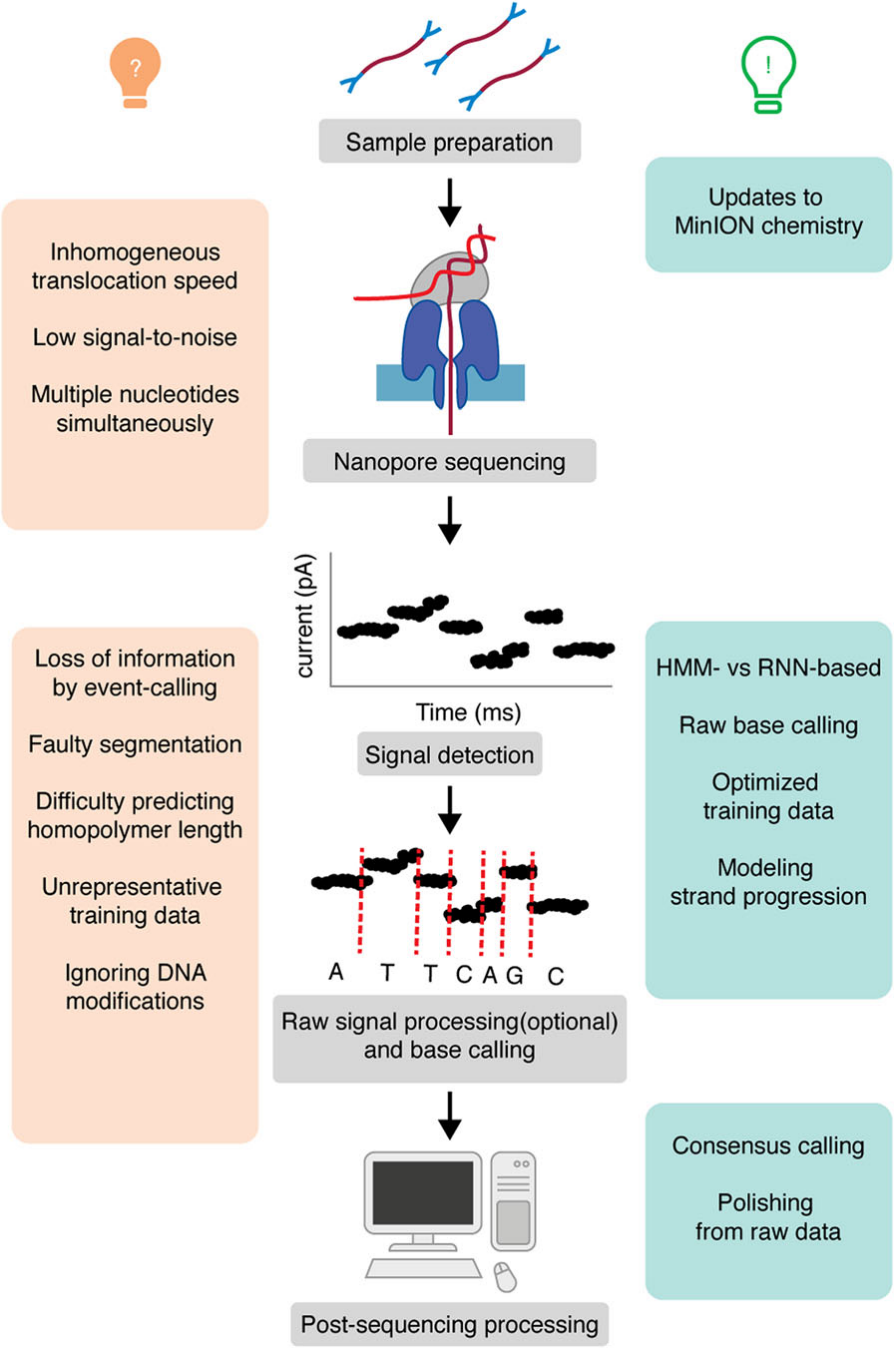
Overview of MinION nanopore sequencing. The left panel shows sources of errors during MinION sequencing and base calling. The right panel shows computational strategies that have been used to improve accuracy. HMM Hidden Markov Model, RNN Recurrent Neural Network

In addition to the chemistry updates released by ONT, computational tools to process the MinION sequencing data and to improve accuracy have been developed, tested, and compared by the scientific community. At the moment, however, an overview of these strategies and a delineation of their contributions is lacking. In this review, we discuss computational approaches to improve the accuracy of nanopore sequencing data by focusing on (i) advances in the computational methods for base calling and (ii) the use of postsequencing correction tools (Fig. 2).

## Sources of errors in nanopore sequencing data

There appear to be two distinct steps at which errors can arise in Oxford Nanopore sequencing data. First, we can reasonably assume that errors can occur during sequencing and thus be inherent to the raw data. In this case, the inherent limitations of the technology result in a low signal-to-noise ratio, making it impossible to determine the underlying DNA sequence. Second, errors could be made in the process of translating the raw electric current signal into a DNA sequence. Here, the information about the DNA sequence is actually present in the data, but shortcomings in the analysis prevent its correct interpretation. The influence of these two steps on the error rate seems to be supported by improvements in accuracy following upgrades in both nanopore chemistry and base-calling software (Fig. 1).

There are several factors in play during sequencing that may contribute to a low signal-to-noise ratio: (i) the structural similarity of the nucleotides; (ii) the simultaneous influence of multiple nucleotides on the signal [23]; (iii) the nonuniform speed at which nucleotides pass through the pore [24–26]; and (iv) the fact that the signal does not change within homopolymers [26] (Fig. 2).

In earlier MinION nanopores (R7, 7.3), the raw current signal was mainly influenced by five or six nucleotides that occupied the pore at any given time point. One measurement thus corresponds to 2048 or 4096 possible k-mers. In the latest pores (R9, 9.4), ONT reports that the three central nucleotides mainly determine the signal, with a smaller influence from more distal nucleotides within the pore [23]. When more nucleotides reside in the pore, one measurement can correspond to even more k-mers and thus more unique signal levels are required to differentiate between them. Consequently, it is more difficult to achieve good signal-to-noise ratios for pores that are influenced by long k-mers compared with those that are occupied by shorter k-mers. Moreover, nucleotides may harbor chemical modifications, such as methyl groups, that affect the signal and effectively increase the number of unique signal levels.

In order to improve signal robustness, the k-mers have to reside within the pore long enough to differentiate signal from noise. The speed at which DNA translocates through a pore under the influence of an electric potential alone is too high to allow reliable detection of each signal [27]. Therefore, Oxford Nanopore chemistry involves the attachment of a motor protein to the DNA, which slows down the translocation and improves the quality of the signal [24, 25]. Nevertheless, despite a reduced translocation speed, it is difficult to detect the transition between two identical k-mers, complicating the detection of homopolymers that are longer than the k-mer. One way to tackle the problem is to infer homopolymer length from the duration of the measured signal. Problematically, the translocation speed of motor proteins is generally nonuniform, disrupting the relationship between homopolymer length and detection time [24–26], a problem that has also been reported by ONT [23]. Consequently, many deletion errors in MinION reads occur in homopolymers [3, 5, 28]. For example, one study reported a 2.6-fold increase in deletion errors for sequences that overlap homopolymers [5].

Errors that arise during signal interpretation, on the other hand, may result from heuristics in the algorithm necessary to bring down the computational costs. For instance, some of the base-calling algorithms assume that two consecutive k-mers at most may be undetected [29], even though larger skips can occur. In addition, the performance of the base callers is influenced by the datasets that are used to train the parameters of the model [13, 30] (Table 1). Biases in the training data—such as type of species or the balance between amplified and nascent DNA (which may contain base modifications such as methylation)—could thus result in errors when applying the resulting parameters to new data.

**Table 1:** Explanation of technical terms

Term	Description	Reference(s)
Beam search	A heuristic search algorithm. In Chiron, the beam search decoder with beam width *W* maintains a list of the *W* most probable sequences up to position *i* and constructs the probabilities of all possible sequence extensions for *i* + 1.	[32]
Connectionist Temporal Classification (CTC) decoder	A type of neural network output and scoring for labeling sequence data with RNNs. It does not require presegmented training data and postprocessed outputs.	[56]
Convolutional Neural Network (CNN)	A type of neural network often used for image analysis. It can recognize patterns by applying different filters to an image.	[57]
Forward algorithm	An algorithm that computes the probability *P*(*x*) of a sequence *x* given a certain HMM.	[58]
Hidden Markov Model (HMM)	A stochastic model that models a sequence of unobserved events underlying a sequence of observations. HMMs assume that an event only depends on the previous event.	[58, 59]
Long-short-term memory (LSTM) unit	A type of RNN that can be used as a building block in bigger networks. It has specific input, output, and forgot gates that allow it to retain or discard information that was passed on from a previous state.	[60, 61]
Partial Order Alignment (POA) graph	A graph representation of a multiple alignment that allows each base in the alignment to have multiple predecessors. Different paths through the graph represent different alignments.	[62]
Recurrent Neural Network (RNN)	A type of neural network that takes information passed on from previous states into account.	[63]
Training data	A dataset that is used to optimize (i.e., train) the parameters of a model. Training is required for both HMMs and RNNs. The training dataset thus determines the performance of the model.	[58, 63]
Viterbi decoding	An algorithm that finds the most likely sequence of events given a certain HMM.	[58]

## Defining read accuracy and error rate

To get a good view of how technological developments impact the accuracy of MinION sequencing data, clear definitions of accuracy and error rate are essential. A wide range of definitions has been used throughout recent publications. For accuracy, these definitions include percent identity to a reference sequence relative to read length [31], alignment length [5, 9, 13, 28], and reference length [32]. Equivalent definitions for error rate are used. Unfortunately, the formulas and tools used to calculate these metrics are often not clearly stated. Probably the most commonly used definition of read accuracy is the percentage of bases in a segment of a read that match with a reference relative to the length of the readsegment–reference alignment:

$$ accuracy=\frac{matches}{matches+ mismatches+\sum \left( length\left( insertions\in read\right)\right)+\sum \left( length\left( deletions\in read\right)\right)}\ast 100\%. $$

Concordantly, the error rate would constitute the percentage of unmatched bases in the alignment and can be subdivided in substitution, insertion, and deletion rates:

$$ error\ rate=\frac{mismatches+\sum \left( length\left( insertions\in read\right)\right)+\sum \left( length\left( deletions\in read\right)\right)}{matches+ mismatches+\sum \left( length\left( insertions\in read\right)\right)+\sum \left( length\left( deletions\in read\right)\right)}\ast 100\% $$

$$ =\frac{errors}{alignmentlength}\ast 100\% $$

It is difficult to measure the impact of technological developments on data quality on the basis of literature reports as there are differences between publications in the ways that the accuracy and error rate are reported. Many researchers report the average read accuracy, whereas others report the median or provide a distribution. A second complicating factor in the comparison of read accuracies is that they depend directly on the performance of the alignment algorithm. Different alignment tools may result in different reported accuracies [33], although they have been reported to yield similar results [3, 28].

Finally, often only a subset of the reads is used to calculate the accuracy and error rate. After reads have been base called, they are divided into high-quality (pass) reads, low-quality (fail) reads, and a subset of reads that cannot be base called. It is not always clear whether pass reads or both pass and fail reads are used to calculate the accuracy of the data. Moreover, the calculated accuracy does generally not take into account the reads that could not be aligned. These filtering steps have a direct impact on the reported accuracy, as there is a trade-off between the accuracy and the yield of the run, i.e., the fraction of reads that are considered as useful data. In order to enable better comparisons of accuracy, it would be advisable to report the accuracy along with the yield of the run, or to report the equivalent of a precision–recall curve in which the accuracy for a range of yields is plotted.

In this review, the mean accuracies of 1D pass reads are reported unless stated otherwise. Figure 1 shows some of the accuracies that have been reported in the literature over the past 3 years, as well as important updates in nanopore chemistry and base-calling algorithms. Owing to the difficulties listed above, there are inconsistencies between the methods by which the accuracies have been obtained. Nevertheless, when combined, these data show a clear trend of improved accuracy since the release of the MinION.

## Base calling

For the current MinION chemistry, ONT reports that single DNA strands are pulled through the pore at an average speed of 450 bp/s, while the electric current is sampled at a frequency of 4 kHz [34]. This means that there are on average nine discrete measurements per k-mer, although the number varies because of the fluctuating translocation speed of the motor protein. In order to translate this raw electric current signal to a DNA sequence, sophisticated base-calling software is required.

In the early days of MinION, base calling was performed by the cloud-based EPI2ME platform provided by Metrichor Ltd., but this feature was discontinued in March 2017. In August 2016, base calling became available in the software program MinKNOW, which runs on the local machine connected to the sequencer to monitor and control MinION sequencing. In addition to the MinKNOW integrated base caller, ONT now offers several other base-calling programs, including the command-line base caller Albacore, and the research base callers Nanonet and Scrappie which have mainly been used as a testing ground for new features. In addition to the ONT base callers, several independent base callers have been developed by researchers in the past 2 years, including Nanocall [29], DeepNano [35], Chiron [32], and BasecRAWller [30]. These ONT base callers have rapidly evolved: Albacore alone was updated at least 12 times between January and September 2017.

The rapid succession and improvement of base callers demonstrates that their performance is an important determinant in the quality of the base pair sequence that is retrieved from the raw signal. In this section, we discuss different approaches to base calling and the most notable improvements that have been made in recent years.

### Hidden Markov models versus recurrent neural networks

To deal with the oversampling, the initial MinION base callers required segmentation of the raw signals into discrete events before base identification. This process reduced the size of the input dataset and combined the redundant measurements into a supposedly more reliable, event-based signal. According to ONT, MinKNOW (up to v1.9) performed segmentation by calculating *t* statistics over two pairs of adjacent sliding windows in the raw signal [19]. These statistics were then combined to determine event boundaries. For each event, the mean, standard deviation, and duration of the raw signal were reported and used for further base calling. The resulting sequences of events are often referred to as ‘squiggles’. To interpret the sequence of events, MinKNOW offers pore models and scaling parameters. The pore models provide distributions of the mean signal and standard deviations that can be expected for each k-mer, while the scaling parameters help to correct for differences in signal that may occur between different wells or over the course of a sequencing run [36].

**Fig. 3:**
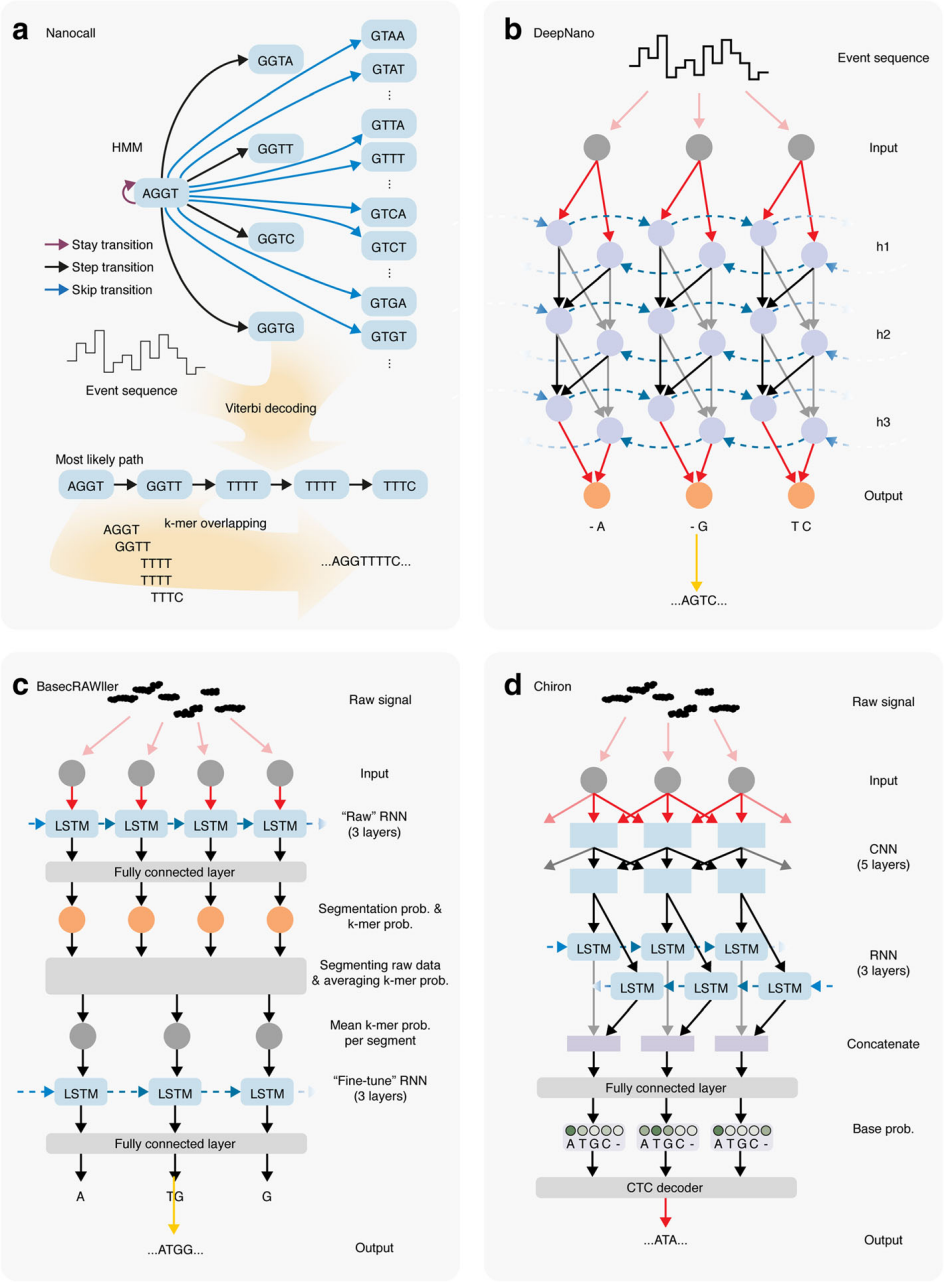
Schematic overview of the algorithms underlying nanopore base callers. **a** Nanocall uses a Hidden Markov Model (HMM) for base calling. **b** DeepNano was the first base caller to use Recurrent Neural Networks (RNN). h1–h3 represent three hidden layers in the RNN. **c** BasecRAWller uses two RNNs, one to segment the raw measurements and one to infer k-mer probabilities. **d** Chiron makes use of a Convolutional Neural Network (CNN) to detect patterns in the data, followed by an RNN to predict k-mer probabilities, which are evaluated by a Connectionist Temporal Classification (CTC) decoder. LSTM long-short-term memory

The first generations of ONT base callers used Hidden Markov Models (HMMs) (Table 1) to predict the DNA sequence on the basis of the event data, pore models, and scaling parameters. The first open-source base caller, Nanocall, employed the same principle [29] (Fig. 3a). In the Nanocall HMM, the hidden states represent all possible k-mers with emission probabilities that are based on the pore models. The transition probabilities, on the other hand, are determined on the basis of a training dataset (Table 1). They mirror the possible event transitions in which a consecutive event can refer to a k-mer shifted by one position in the DNA sequence (*step*), a k-mer shifted by more than one position (*skip*), or the same k-mer (*stay*). To speed up computation, skips with a size larger than one are not allowed in the HMM. During base calling, the most probable path through the hidden states is calculated by Viterbi decoding (Table 1). The path is converted to the final base sequence by merging the sequence corresponding to two consecutive states according to their maximal overlap. The consequence of this heuristic is that homopolymer repeats of a length greater than the size of the k-mer cannot be detected.

Soon after the publication of Nanocall, the first version of the base caller DeepNano was published [35]. Rather than using HMMs, DeepNano uses Recurrent Neural Networks (RNNs) (Table 1), which do not explicitly rely on k-mer length and are able to take longer range information (i.e., >k bp) into account. Since information about the DNA sequence is contained in events both upstream and downstream of the current event, DeepNano uses a bidirectional RNN that makes predictions for events in each direction and combines the two predictions for each event in the next layer of the neural network (Fig. 3b).

In terms of performance, the RNN-based DeepNano achieves a substantial improvement over the HMM-based callers. On R7.3 data, Metrichor called 1D reads with an accuracy of 70–71% and Nanocall with an accuracy of 68%, whereas DeepNano reached accuracies of up to 77% [29, 35]. For 2D reads, this difference was less pronounced, with Metrichor reaching an accuracy of 87% and DeepNano an accuracy of 89%. Nanocall does not provide an option to call 2D reads.

Before the final version of the DeepNano paper was published, ONT released their own RNN-based base caller, Nanonet. The general principle is similar to that of DeepNano. Nanonet employs bidirectional long-short-term memory (LSTM) units (Table 1) to utilize information from both upstream and downstream states. In the final publication of DeepNano, the authors compare the accuracy of the two RNN-based base callers on an *E. coli* dataset produced with R9 chemistry and show that they perform similarly on 1D reads (DeepNano ~ 81%, Nanonet ~ 83%) [35]. Given the superior performance of RNN base callers when compared with HMM base callers, algorithms similar to those of Nanonet have been adopted in newer versions of the MinKNOW base caller and in all versions of the ONT base callers Albacore and Scrappie.

### Base calling using raw signal

Although initial versions of early base callers used the segmented event data provided by MinKNOW as input to determine the DNA sequence, current base callers use raw current signal as input. The Scrappie base caller (which is available through a developer license from ONT) was the first base caller to employ raw current signal. In addition, the recent introduction of BasecRAWller provides a well-documented base-calling method for raw nanopore data [30].

BasecRAWller employs two separate RNNs (Fig. 3c). The first RNN uses each measurement point to predict the probability that a signal corresponds to a new k-mer and the probability of the k-mer identity simultaneously. On the basis of the probabilities of k-mer transitions, the raw signal is segmented and the k-mer probabilities are averaged over the segments. These probabilities are then fed into the second RNN, which predicts the final DNA sequence. Importantly, both RNNs use LSTMs to pass contextual information forward but not backward, making the technology fast enough to base call reads as they pass through the pore. However, the increase in processing speed comes at the cost of accuracy.

Although BasecRAWller uses the raw current signal rather than the events detected by MinKNOW, it still performs an internal segmentation step after the first RNN. The recently introduced base caller Chiron, on the other hand, is capable of translating the raw signal into a DNA sequence without an intermediate segmentation step [32] (Fig. 3d). In Chiron, the raw data are first fed through a Convolutional Neural Network (CNN) (Table 1), which detects local structures in the signal. The output of the CNN is used as input for an RNN that makes use of bidirectional LSTMs. The RNN outputs base call probabilities that are evaluated by a Connectionist Temporal Classification (CTC) decoder (Table 1) and are converted to a sequence of bases by a beam search algorithm (Table 1). Despite being trained on limited amounts of data, Chiron had similar accuracies compared with Albacore v2.0.1 and outperformed the segmentation-based Albacore v1.1 This was also the case for human sequencing data, even though Chiron was trained on nonhuman data only.

Around the same time that the first version of Chiron was published, ONT transitioned to raw base calling in a new update of Albacore (v2.0.1). Internal testing performed by ONT showed that raw base calling improves the modal read accuracy by 1% over that achieved by event-based base calling [37]. The increase in accuracy is due to the fact that mistakes made during segmentation are hard to correct later on, as information is lost when the raw data are reduced to mean, deviation, and duration values alone.

### Training of base callers for base composition and modifications

An important aspect of the current base callers is that they require training to optimize the parameters of the HMM or RNN. Consequently, the nature of the training dataset is crucial in determining base caller performance on sequencing data from different biological samples. Depending on the source of the DNA and the sample preparation, sequencing datasets may have different characteristics, such as different base composition or base modifications, that should be sufficiently accounted for during training.

Genome structure may vary between species with regard to GC content [38], codon usage [39], and nature of DNA modifications. While testing BasecRAWller, it became apparent that using an *E. coli* training set resulted in much higher accuracies on new *E. coli* sequencing data than on human data [30]. Interestingly, when human training data were used, the accuracies for *E. coli* and human data were more comparable. In another comparison of base callers, Scrappie v1.1.1 achieved a read accuracy that was more than 2% higher than that achieved by Scrappie v1.1.0 for a bacterial *Klebsiella pneumoniae* dataset [13]. This improvement in accuracy can most probably be attributed to the fact that v1.1.1 was trained on a mixed set of genomes, whereas v1.1.0 was trained on human data only. Together, these observations indicate that the nature and originating species of the training data plays an important role in base caller performance. As for now, it remains unclear whether the broad applicability of training data depends on their k-mer diversity or on similarity between species. The latter case may be problematic because it would mean that suboptimal performance can be expected for species for which no training data are available.

As the MinION effectively probes nucleotide structure, chemical modifications such as methyl groups influence the signal. If such DNA modifications are not represented in the training data, they may result in erroneous base calls. This is one of the reasons why a substantial difference in base quality is observed between sequence runs that use nascent DNA rather than PCR-amplified DNA. The problem may be solved either by training the parameters to recognize modified bases and to call them as their canonical nucleotide (e.g., 5-mC as C), or by treating them as distinct bases. At the moment, the option to take DNA modifications into account in the base calling has not yet been incorporated into the ONT base callers [37]. Using the open-source base caller Nanonet, however, efforts are being made by the ONT community to include modified bases in the RNN [40]. The feasibility of calling base modifications from MinION data has already been demonstrated by the fact that DNA modifications have successfully been derived for nascent DNA sequencing data after base calling [41–44].

### Modeling strand progression

The detection of homopolymers with nanopores is more challenging because consecutive k-mers are identical. As the segmentation step often resulted in more events than actual bases, initial base callers assumed that identical signals were the result of stalling in the pore rather than signals originating from a homopolymer. In order to improve homopolymer calling, a so-called transducer has recently been included in the ONT base caller Scrappie. According to ONT, the transducer enables the separate prediction of k-mer identity and movement [45]. Early results indicate that Scrappie is indeed more successful at calling homopolymers than ONT base callers without the transducer: Scrappie could call homopolymers of up to 20 bases correctly, whereas Nanonet and the Metrichor base caller consistently predicted a length of ∼ 5 bases for all homopolymers with a length greater than 5 bases [3]. The transducer has subsequently been adopted in both the MinKNOW base caller (as of v1.6.11) and in Albacore (as of v1.0.1).

## Postsequencing correction

Developments in nanopore chemistry and base-calling algorithms have resulted in a considerable increase in read accuracy over the past few years. Depending on the nature of the sample and the desired application, further improvements in accuracy can be made by performing postsequencing correction. Several correction algorithms are available that make use of three (not mutually exclusive) approaches: (i) consensus finding, (ii) polishing based on raw data, and (iii) hybrid error correction. The last uses short-read data to correct MinION reads or an assembly that is based on MinION reads [9]. As hybrid tools do not use information inherent to nanopore data to improve accuracy, they are not included in this review.

### Consensus calling

The generation of multiple alignments of nanopore reads and the extraction of consensus sequences has the potential to eliminate all random errors, leaving only systematic errors that are introduced during sequencing or base calling. As long reads are very useful in genome assembly, several tools that call consensus sequences from MinION data have been developed specifically for this purpose. These postsequencing tools generally perform consensus calling either on reads or on genome assemblies by constructing Partial Order Alignment (POA) graphs (Table 1).

Genome assembly tools that implement POA graphs for nanopore consensus calling and read correction include Nanocorrect [15], Racon [46], and Canu [14]. Nanocorrect was shown to improve read accuracy from 80.5 to 97.7% based on 29× coverage [15]. Despite this success, Nanocorrect has been deprecated because it is rather slow, and better-performing assembly pipelines have become available [47]. Racon can either be used for read correction or paired with genome assemblers that do not perform prior read correction [46]. 2D R7.3 reads with a coverage of 54× and a median accuracy of 89.8% were corrected to an accuracy level of 99.25%. When Racon was used to improve genome assemblies computed with Miniasm [16], the assembly accuracy varied between 97.7% (30× coverage) and 99.32% (54× coverage) [46]. Finally, Canu is a genome assembly tool that incorporates POA graphs for read correction [14]. On the same datasets as those used for genome assembly with Miniasm+Racon, Canu obtains accuracies ranging between 96.87% (30× coverage) and 98.61% (54× coverage) [46].

The consensus tools developed for genome assembly rely on the assumption that all reads in a dataset are derived from one homogeneous genetic source. In the case of mixed samples or polyploidy, consensus calling should only be performed on reads known to stem from the same source. Multiple reads derived from the same genetic material can be obtained by experimental methods such as INC-seq, in which tandem copies of a target sequence are generated with circular amplification prior to sequencing [48, 49].

### Consensus polishing from raw signal

Although each base-called read represents the most likely prediction of the underlying nucleotide sequence based on the observed event sequence or raw signal, the raw data retains more information than is represented in the final sequence. For this reason, squiggle or raw data describing overlapping reads can be combined to assess and correct a proposed assembly sequence. Loman et al. [15] used this principle in Nanopolish, a tool aimed at improving (i.e., polishing) draft genome assemblies that were based on nanopore event data.

Nanopolish starts by mapping the uncorrected reads to the draft assembly, which represents the initial consensus that is based on all base-called reads. Subsequently, the assembly is divided into overlapping segments that can be processed in parallel. Within each segment, the aligned reads are reverted back into their squiggle counterparts that were observed during sequencing, defined by the mean current per k-mer. A series of slightly altered sequences is then proposed and their probabilities given the set of event sequences are compared. These probabilities are obtained by applying the Forward algorithm (Table 1) on an HMM that is structured similarly to the Nanocall HMM. The sequence with the highest probability replaces the segment of the initial assembly and a new set of modifications is proposed. The process stops after a set number of iterations or when the consensus no longer changes. Finally, all overlapping segments are combined into the final assembly.

Nanopolish has evolved since its initial release and is compatible with the newest sequencing kits and base-calling tools. In addition, Nanopolish has recently obtained a new functionality that allows it to detect and call methylated bases [42]. An online comparison of base callers shows that the methylation-aware option reduces errors in assemblies that are based on nascent DNA sequencing data, since chemical modifications affect the raw signal [13].

Nanopolish is now commonly used to finalize genome assemblies that are based on nanopore data. In general, the application of Nanopolish results in improvements of around 0.1–0.5% [13, 15], but in some cases it may result in an increase of > 2% [13, 14]. Interestingly, draft assemblies that are constructed using base callers that have vastly different performances can be polished to a similar level of accuracy by Nanopolish [13], implying that base-caller performance may not be the limiting factor in genome assembly applications.

## Discussion and outlook

Nanopore sequencing offers the possibility of producing long reads from single DNA molecules in real time and has the potential to open up the field of sequencing to many new applications. Since its initial release, the technology marketed by ONT in the form of the MinION has suffered from a high error rate. Thanks to several updates in chemistry and software tools, the raw read accuracy has already increased from < 60% to > 85%. At the moment, data produced by the MinION are sufficiently accurate to create consensus (genome) assemblies of > 99% accuracy. In spite of these feats, however, the current nanopore read accuracy still limits robust calling of SNVs and indels, especially in complex samples such as tumors.

Here, we have discussed several of the computational strategies that have been used to improve read accuracy since the release of the MinION in 2014. With regard to base calling, the most notable developments include the switch from HMMs to RNNs and the use of raw current signal instead of segmented signal as input. Moreover, the first steps towards modeling strand progression through the pore in order to achieve better estimates of homopolymer length have shown promising results. Other notable innovations include the use of raw current signal to improve consensus sequences, as implemented by Nanopolish. Recently, ONT released an early version of their own postassembly polishing tool, Medaka (https://nanoporetech.github.io/medaka/index.html), indicating that this is an active field of research that will probably lead to further improvements in postsequencing correction.

Despite the many different analytical tools that have been developed over the past few years, it remains difficult to establish all determinants of read accuracy and error rate clearly. In part, this ambiguity can be attributed to the fact that variable definitions of accuracy and error rate are being used, definitions are not always clearly stated, and results are reported inconsistently (for example, some studies report the median, mean, or a distribution of the error or accuracy). Moreover, accuracy is usually calculated only for a subset of the data, that is only for alignable, high-quality reads. In order to obtain a full picture of developments in read accuracy, the percentage of alignable reads and the accuracy over all aligned reads needs to be reported. Problematically, this is only possible when a high-quality reference genome is available, which may not be the case for many important applications of the MinION. Nevertheless, consistent reports of accuracy will be important to show which contributions are most successful in improving the error rate of nanopore sequencing. The blog post by Wick et al. [13] is a prime example of how the community can make valuable contributions by systematically comparing computational strategies.

To date, improvements in read accuracy have been achieved through four general strategies: (i) improvement of the pore itself (e.g., the evolution from R6 to R9); (ii) the use of library preparation methods that allow for a piece of DNA to be read multiple times (e.g., 2D and 1D^2^ sequencing); (iii) innovations in base-calling algorithms (e.g., from HMM to RNN); and (iv) the development of postsequencing correction tools (e.g., Nanopolish). Error rates are likely to decrease further with improved pore chemistries, innovative library preparation methods, and better software. For example, rolling circle amplification can be used to create tandem copies of DNA templates [48], and this approach is especially suitable for complex samples where sequence information pertaining to a specific allele, cell, or species is thus linked. Meanwhile, base callers have been developing at a rapid pace over recent years, with continuous improvement evident. An important future avenue for further improvement may be the use of species- and library preparation-specific training data in the base-calling algorithm.

With these prospects in mind, the question arises: is there an inherent ceiling to nanopore read accuracy? It is certainly true that accuracy has improved markedly over the past few years, but sequences with low complexity such as homopolymers are still notoriously difficult to call accurately. Reassuringly, the recent version of the ONT base caller Scrappie demonstrates that homopolymer calling is at least not inherently impossible for the MinION. Nevertheless, it is unlikely that systematic errors can be abolished completely. This also seems to be acknowledged by ONT, who are actively working on improved pore designs. A particularly promising direction of research is to use pores with multiple recognition sites that are separated by a distance of ~ 15 bp, which would allow variable signal to be detected within homopolymers of up to 30 bp [37].

By clearing the last few percent of errors, these developments may in the near future allow nanopore sequencing to begin to compete seriously with short-read platforms in the robust detection of SNVs and indels in complex samples, and in many more applications.

## Additional file

Additional file 1: Supplemental Figure S1 and Table S1. (DOCX 75 kb)
