# Supplementary material for: From squiggle to basepair: computational approaches for improving nanopore sequencing read accuracy
Source: Genome Biol. 2018 Jul 13;19:90. doi: 10.1186/s13059-018-1462-9 (PMC6045860; doi:10.1186/s13059-018-1462-9)
Supplement: Supplementary file 1 — Supplemental Figure S1 and Table S1. (DOCX 75 kb) [file 13059_2018_1462_MOESM1_ESM.docx]

**Additional file 1**

From squiggle to basepair: computational approaches for improving nanopore sequencing read accuracy

Franka J Rang^1^, Wigard P Kloosterman^*1^ and Jeroen de Ridder^*1^

^1^Department of Genetics, Center for Molecular Medicine, University Medical Center Utrecht, Utrecht University, 3584 CG, Utrecht, The Netherlands

*Correspondence: W.Kloosterman@umcutrecht.nl, J.deridder-4@umcutrecht.nl

**Table S1:** **Comparison of long-read (MinION, PacBio Sequel) and short-read (Illumina MiSeq) sequencing platforms.** All prices are approximate and reported in US dollars. Data are largely based on information provided by manufacturers: [[1–4]](https://paperpile.com/c/hmMdcj/kzFlV+L5afV+tzrnf+TLDbl) (MinION), [[5–8]](https://paperpile.com/c/hmMdcj/CN6G+Y7eu+Ek1p+wGqn) (Sequel), and [[9–11]](https://paperpile.com/c/hmMdcj/ebSOy+NvNhS+mqXYC) (MiSeq). *The read lengths provided by the MinION are variable and limited by the size of the input DNA. **^§^**The actual number of reads is variable and depends on the selected read length. Both the MinION and Sequel can read the same DNA fragment multiple times, using the accumulated sequencing information to obtain an improved consensus sequence. ^#^Prices based on flow cell bulk discount. NA, not applicable.

|  | **ONT MinION** | **PacBio Sequel** | **Illumina MiSeq** |
| --- | --- | --- | --- |
| **Read accuracy** | 85 % (1D) | 86 % (single-pass) | *>*99 % |
| **Read accuracy (consensus)** | 97 % (1D^2^) | *>*99 % (15x circular consensus sequencing) | NA |
| **Mean read length** | 1–50 kb* | 1–15 kb | NA |
| **N50 read length** | 1–100 kb* | 1–30 kb | NA |
| **Max. read length** | *>*1 Mb* | *>*90 kb | 2 × 300 bp |
| **Reads per flow cell**^§^ | Up to a few million | Up to 365 thousand | 25 million |
| **Typical data output** | 1Gb–20 Gb | 5–8 Gb | 0.3–15 Gb |
| **Amplification required** | No | No | No |
| **Library preparation time** | 10 min (rapid kit) | 3–6 hours | 90 min (Nextera) |
| **Run time** | 0.5–48 hours | 0.5–10 hours | 5–55 hours |
| **Data acquisition timing** | Real-time | Real-time | Post-sequencing |
| **DNA modification detection** | Yes | Yes | No |
| **Size (W** × **D** × **H)** | 10 × 3 × 2 cm | 92.7 × 86.4 × 167.6 cm | 68.6 × 56.5 × 52.3 cm |
| **Weight** | 90 g | 354 kg | 57.2 kg |
| **Sequencing location** | Anywhere | Lab | Lab |
| **Capital investment** | $1000 | $350,000 | $125,000 |
| **Cost per run** | $700^#^ | $850 | $1400 |


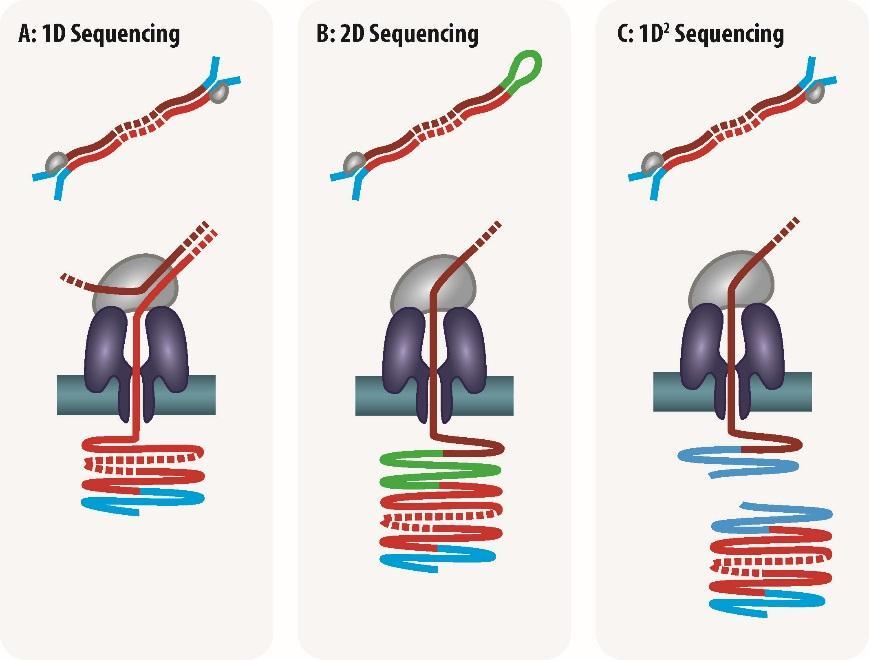


**Figure S1:** **MinION chemistries for sequencing of double-stranded DNA.** **a.** During 1D sequencing, only one of the two strands of a double-stranded DNA (dsDNA) molecule is read, starting from an adapter (blue) that is ligated to the dsDNA molecule. **b.** In 2D sequencing, which is currently deprecated, a hairpin adapter (green) is used to connect the two strands, allowing both to be sequenced. **c.** 1D^2^ chemistry promotes the sequencing of the second strand (also denoted as the complement) by keeping this strand in proximity to the pore and increasing the chance of its capture after the first strand (template) has passed through the pore

**Additional references**

[1. Jain M, Koren S, Miga KH, Quick J, Rand AC, Sasani TA, et al. Nanopore sequencing and assembly of a human genome with ultra-long reads. Nat Biotechnol. 2018;36:338–45.](http://paperpile.com/b/hmMdcj/kzFlV)

[2. Oxford Nanopore Technologies Store.](http://paperpile.com/b/hmMdcj/L5afV) <https://store.nanoporetech.com/> Accessed 20 Apr 2018.

[3. Oxford Nanopore Technologies. MinION.](http://paperpile.com/b/hmMdcj/tzrnf) <https://nanoporetech.com/products/minion>. Accessed 20 Apr 2018.

[4. Oxford Nanopore Technologies. 1D squared kit available in the store: boost accuracy, simple prep.](http://paperpile.com/b/hmMdcj/TLDbl) <https://nanoporetech.com/about-us/news/1d-squared-kit-available-store-boost-accuracy-simple-prep>. Accessed 20 Apr 2018.

[5. Pacific Biosciences.](http://paperpile.com/b/hmMdcj/CN6G) AllSeq. The sequencing marketplace. <http://allseq.com/knowledge-bank/sequencing-platforms/pacific-biosciences/>. Accessed 20 Apr 2018.

[6. PacBio. Revolutionize genomics with SMRT® sequencing.](http://paperpile.com/b/hmMdcj/Y7eu)  https://www.pacb.com/wp-content/uploads/SMRT-Sequencing-Brochure-Revolutionize-genomics-with-SMRT-Sequencing.pdf. Accessed 20 Apr 2018.

[7. Eid J, Fehr A, Gray J, Luong K, Lyle J, Otto G, et al. Real-time DNA sequencing from single polymerase molecules. Science. 2009;323:133–8.](http://paperpile.com/b/hmMdcj/Ek1p)

[8. PacBio. SMRT sequencing: read lengths.](http://paperpile.com/b/hmMdcj/wGqn) <https://www.pacb.com/smrt-science/smrt-sequencing/read-lengths/>. Accessed 24 Apr 2018.

[9. Illumina. Focused power on](http://paperpile.com/b/hmMdcj/ebSOy) the MiSeq System. <https://www.illumina.com/systems/sequencing-platforms/miseq.html>. Accessed 20 Apr 2018.

[10. Illumina. AllSeq.](http://paperpile.com/b/hmMdcj/NvNhS) <http://allseq.com/knowledge-bank/sequencing-platforms/illumina/>. Accessed 20 Apr 2018.

[11. Illumina. Nextera DNA Library Prep Kit.](http://paperpile.com/b/hmMdcj/mqXYC) <https://www.illumina.com/products/by-type/sequencing-kits/library-prep-kits/nextera-dna.html>. Accessed 20 Apr 2018.
